# Is It Time to Switch from Conventional Coombs Crossmatching to the Type and Screen Protocol?

**DOI:** 10.21315/mjms2023.30.2.11

**Published:** 2023-04-18

**Authors:** Nithya M Baiju, Aboobacker Mohamed Rafi, Nittin Henry, Ramesh Bhaskaran, Susheela Jacob Innah, Athira Sasidharan

**Affiliations:** 1 Department of Transfusion Medicine, Jubilee Mission Medical College and Research Institute, Thrissur, Kerala, India; 2 Department of Transfusion Medicine, MVR Cancer Center, Calicut, India

**Keywords:** compatibility testing, Type and Screen protocol, Coombs crossmatching

## Abstract

**Background:**

ABO grouping, Rh typing and crossmatching are routinely done as part of pre-transfusion testing. The Type and Screen (T&S) protocol has been used in developed countries to ensure the survival of transfused red cells. In this study, we compared the safety, costs and turnaround times (TATs) of the T&S protocol and the conventional pre-transfusion testing protocol for patients who had been scheduled for elective obstetrical or gynaecological procedures.

**Methods:**

This observational study was conducted in three phases at the Department of Transfusion Medicine, Jubilee Mission Medical College and Research Institute, Kerala, India and involved 1,800 patients from the Department of Obstetrics and Gynaecology, Jubilee Mission Medical College & Research Institute, Kerala, India over the course of 2 years. Phase I involved the traditional pre-transfusion testing and crossmatching of 150 patients. Phase II involved the use of the T&S protocol on 150 patients. Phase III involved the use of both the traditional and T&S protocols on 1,500 patients without considering the results of each protocol. The safety, costs and TATs of both protocols were compared.

**Results:**

In this study, the T&S protocol provided a safety 100% level when compared to the traditional protocol. The T&S protocol detected unexpected antibodies in 0.4% of cases, which would have gone unnoticed otherwise, demonstrating its usefulness. There was no significant difference in cost between the traditional crossmatching and T&S protocols. We discovered that using only the T&S protocol can save technologists 30% of their time.

**Conclusion:**

Implementing the T&S protocol as a pre-transfusion testing procedure can help improve hospital transfusion practices by supplying blood quickly and safely. Coombs crossmatching remains more of a tradition than a necessity.

## Introduction

Pre-transfusion testing mainly includes ABO and Rh typing, screening for unexpected antibodies and crossmatching between patients and donor units (1). ABO and Rh typing involve the forward and reverse typing of ABO and Rh antigens in both the donor and the recipient. Crossmatching checks the compatibility between the donor blood component and the recipient. The crossmatch procedure usually takes about 45 min (2).

In 1984, the American Association of Blood Banks recommended abbreviated crossmatching as a replacement for full crossmatching in patients with negative antibody screens (3). The possibilities of a clinically significant red cell antibody being undetected in a patient with negative antibody screens are 1–4/10,000 (4–6). These recommendations led to the development of the Type and Screen (T&S) protocol (7, 8).

The primary objective of this study was to compare the safety of the T&S protocol and Coombs crossmatching for compatibility testing in our tertiary care centre. The T&S protocol, rather than crossmatching, has been adopted solely for transfusion practices by many hospitals in developed countries. This protocol has proven to be effective without compromising patient safety (9). It also allows for the optimal use of donor blood because it is neither withheld from inventories (by being crossmatched) nor reserved for patients who may not actually require it, which enhances inventory management (10).

It appears that physicians frequently order more units of crossmatched blood than is required and that this practice is based on habit (11, 12). The ensuing crossmatching is unnecessary, costly and wasteful. The crossmatched blood is reserved for the patient for 72 h, endangering the blood stock required for emergency use. This reservation also results in the outdating of units. However, under the T&S protocol, the blood is not reserved and is made readily available on request (13).

Comparing the T&S protocol with Coombs crossmatching will help to determine which of the two procedures can be abbreviated while maintaining patient safety, which will improve hospital transfusion practices. This study was conducted because of the limited literature on the safety of these two pre-transfusion testing procedures among the South Indian population. This comparative study is of clinical relevance in any tertiary care centre because of its potential effects on improving inventory management and resource utilisation.

## Methods

This observational study was conducted at the Department of Immunohematology and Transfusion Medicine, Jubilee Mission Medical College and Research Institute, a tertiary health care centre in Kerala, for 2 years. The study protocol was approved by the Institutional Ethics Committee. This study was implemented for the pre-transfusion testing requests received from the Department of Obstetrics and Gynaecology for elective surgical procedures that were undertaken during the time frame. The study was conducted in three phases.

In phase I, Coombs crossmatching was used to test the compatibility of 150 participants from the Department of Obstetrics and Gynaecology for elective surgical procedures.

In phase II, the T&S protocol was used to test the compatibility of 150 participants from the same department.

In phase III, the T&S protocol was performed on 1,500 participants from the Department of Obstetrics and Gynaecology, followed by Coombs crossmatching of the respective donor units without considering the result of each protocol to determine the safety of both protocols.

The cost and turnaround time (TAT) of both protocols from phases I and II were compared. The safety of both protocols from phase III was compared and analysed.

Inclusion criteria: Patients who were scheduled for elective surgical procedures under the Department of Obstetrics and Gynaecology and for whom a request for crossmatching was received.

Exclusion criteria: Patients who required a massive transfusion protocol and emergency surgical cases were excluded from the study.

Sample type: Ethylenediaminetetraacetic acid (EDTA) tubes were used for blood grouping, cross matching, antibody screening and antibody identification.

### Methodology

Crossmatching, antibody screening and antibody identification were conducted using column agglutination technology (CAT) and antihuman globulin cassettes (anti-IgG) from Ortho Clinical Diagnostics. The low-ionic-strength saline-indirect antiglobulin test technique was performed. Incubation and centrifugation tests were conducted using a semi-automated platform from Ortho Clinical Diagnostics. All the patients in phases II and III were screened for red cell antibodies using three cell panels (SURGISCREEN 1, 2 and 3) from Ortho Clinical Diagnostics. Patients who had positive antibody screens were subjected to antibody identification using 11 cells (RESOLVE panel A). The donors were subjected to antibody screening using single O pooled cells. All the tests were performed according to the departmental standard operating procedure.

### Statistical Analysis

Categorical and quantitative variables data were expressed as frequency (percentage) and mean ± SD, respectively. Comparison of means of quantitative variable between the groups was analysed by using independent *t*-test. Diagnostic statistics such as sensitivity, specificity, positive predictive value, negative predictive value and accuracy have been calculated to assess the safety of T&S protocol when compared to Coombs crossmatch. For all statistical interpretations, *P* < 0.05 value was accepted as being significant. Statistical analysis was performed with statistical software package SPSS, version 21.0.

## Results

A total of 1,800 patient samples for crossmatch from the department of Obstetrics and Gynaecology were enrolled in the study during the 2-year time frame. The mean age of the study population was 41.9 ± 10.2 years old. The mean haemoglobin of the study population was 11.8 ± 1.1 g/dL.

### Safety

The safety of T&S protocol versus Coombs crossmatch was analysed for 1,500 patient samples received for crossmatch from the department of Obstetrics and Gynaecology. T&S protocol and Coombs crossmatch were done independently on the samples without knowing the result of each other. From the 1,500 samples of Phase III, antibody screening was positive in six cases. The rate of alloimmunisation in our study was 0.4% (6/1,500).

Out of the 1,494 T&S negative samples, no sample turned Coombs crossmatch incompatible (Table 1). The study demonstrated safety of T&S method to be 100% (Table 2). It was found to be equally safe.

### Cost and TAT

Cost and TAT of both the protocols of pre-transfusion testing were compared in Phases I & II. There was no significant difference in cost between the Coombs crossmatch and T&S protocol (Table 3). It was found that there was a significant difference in the TAT when T&S protocol was implemented. The mean turnaround time with T&S protocol was 23.8 min compared to the 33 min in the Coombs crossmatch method (Table 4). Thirty percent of the technologist’s time could be saved using the T&S protocol.

## Discussion

Pre-transfusion compatibility testing is a set of procedures and processes to ensure that the selected donor red cells for the recipient will have an acceptable survival when transfused and not lead to clinically significant destruction of those transfused red cells (14, 15). Red blood cell (RBC) alloimmunisation and development of alloantibodies is one of the common risks associated with RBC transfusion (16–19). The rate of alloimmunisation in our study was 0.4%. The study population constituted patients from the department of Obstetrics and Gynaecology. The most common antibody encountered was anti-D (33.3%; 2/6) followed by anti-M (16.6%; 1/6), anti-c (16.6%; 1/6), anti-E (16.6%; 1/6) and anti-Le^a^ (16.6%; 1/6). Prevalence of blood group antigens in Indian donor population by Makroo et al. (20) reported D (93.6%), C (87.0%), c (58.0%), E (20.0%), e (98%), K (3.5%), k (99.97%), Fy^a^ (87.4%), Fy^b^ (57.6%), Jk^a^ (81.5%), Jk^b^ (67.4%), M (88.7%), N (65.4%), S (54.8%) and s (88.7%).The chances of clinically significant antibody being missed in a patient with negative antibody screen are 1–4/10,000. In current study, antibody screening cells (Surgiscreen I, II and III) picked up all the clinically significant antibodies. The usefulness of the T&S was shown through detection of unexpected antibodies in 0.4% (4 out of 1,500) of cases, which would have been missed otherwise with conventional Coombs crossmatch. The study demonstrated that T&S method achieves safety level of 100%. Agrawal et al. (21) had reported 100% concordance between antibody screen and Coombs crossmatch in 45,373 patients. A prospective study done by Heddle et al. (22) concluded that the antiglobulin phase of the crossmatch can be omitted from pre-transfusion testing without putting patients at risk. Comparing the turnaround time of both the pre-transfusion testings, it was found that T&S protocol had a better turnaround time saving approximately 30% of the technologist’s time. The costs of both the protocols were compared and it was found that there is no significant difference. The costs of both the protocols were comparable. In a study done by Masouredis (10) it was stated that the estimated savings from eliminating the antihuman globulin (AHG) phase of a crossmatch is approximately $1.00 in cost and 30% in technologist’s time. Although T&S is a safer alternative to Coombs crossmatch, there are data showing that antibody screen negative cases turned crossmatch incompatible. In a study done by Chaudhary et al. (9), one sample (1 in 12 cases) gave antibody screen negative while Coombs crossmatch was incompatible. However, the study could not establish the specificity of the alloantibody. This may be due to a rare antibody in the patient sera against which the corresponding antigen was not present on the reagent red cell.

When Coombs crossmatch was practiced in the hospital, blood units were reserved for a designated patient for 72 h. If the reserved units had been depleted or exceeded the reservation date, repeat blood sampling and crossmatching would have been required if additional units were needed. A repeat crossmatch required at least another 1 h. The blood stock needed for emergency use was jeopardised as the units are being tied up in the reserve. But under T&S protocol, blood units were no longer reserved for a patient if the results from antibody screen were negative (23). Instead, a validity period is given to an individual for its negative antibody screen status, so that within that time as many units as possible can be issued after performing an abbreviated crossmatch depending on the amount of serum available. For patients who have received a transfusion or who have been pregnant within the preceding 3 months of transfusion or whose history is unknown, the validity period given is 3 days, since antibodies can develop within that time (24). Thus, for three consecutive days, no additional blood sampling is performed, even if repeated transfusions are required. Compatible units can be made available in less than 10 min following an immediate spin crossmatch. Thus, compatible units can be made available within 10 min following T&S protocol. Patients who require a massive transfusion will benefit most from it because as many additional compatible units as required can be issued quickly without the need for taking a new blood sample for repeat crossmatching.

In an Indian study done by Tiwari et al. (25) the safety of immediate spin crossmatch for red blood transfusion in antibody screen negative recipients were established. It was found that 99.7% of the immediate spin (IS) compatible red cell units were also compatible on post-transfusion AHG crossmatch. They concluded in their study that in antibody screen negative patients, immediate spin crossmatch is as safe as AHG crossmatch and can, therefore, replace AHG crossmatch protocol.

From the current study, T&S protocol was proven to be safe, time saving and effective when compared to the Coombs crossmatching. Limited published literatures are available on the safety of T&S protocol with respect to Coombs crossmatching in Indian population, hence further studies need to be done to abbreviate any one of the two procedures.

## Conclusion

In present study, the safety of T&S protocol over Coombs crossmatch was detected to be 100%. The usefulness of T&S protocol was shown through detection of unexpected antibodies in 0.4% of cases, which would have been missed otherwise with Coombs crossmatch. On analysing the turnaround time of both the pretransfusion testings, it was detected that 30% of the technologist’s time could be saved using T&S protocol. The cost was comparable for both the protocols. Henceforth, implementation of T&S protocol as pre-transfusion testing over Coombs crossmatch can help in improving the transfusion practices.

## Figures and Tables

**Table 1:** Comparison of T & S protocol with Coombs crossmatch

T & S	Coombs crossmatch (XM)		

	Incompatible	Compatible	Total
Positive	2	4	6
Negative	0	1,494	1,494

Total	2	1,498	1,500

**Table 2:** Safety parameters of T&S protocol at the tertiary care centre

Parameter	%
**Sensitivity**	100.0
**Specificity**	99.7
**Positive Predictive value**	33.3
**Negative Predictive value**	100.0
**Accuracy**	99.7

**Table 3:** Comparison of cost (in Rupees) based on method

Method	Mean	SD	*N*	*t*	*P*-value
XM	190.7	35.4	150	0.75	0.452
T & S	193.3	25	150		

**Table 4:** Comparison of turnaround time (in minutes) based on method

Method	Mean	SD	*N*	*t*	*P*-value
XM	33	2.1	150		
T&S	23.8	1.3	150	45.65**	0.01

Note:

** Significant at 0.01 level

## References

[1] Chapman JF, Forman K, Kelsey P, Knowles SM, Murphy MF, Wood JK (1996). Guidelines for pre-transfusion compatibility procedures in blood transfusion laboratories. Transfus Med.

[2] Grove-Rasmussen M (1964). Routine compatibility testing: standards of the American Association of Blood Banks as applied to compatibility tests. Transfusion.

[3] American Association of Blood Banks (AABB) (1984). Standards for blood banks and transfusion services.

[4] Oberman HA, Barnes BA, Friedman BA (1978). The risk of abbreviating the major crossmatch in urgent or massive transfusion. Transfusion.

[5] Mintz PD, Haines A, Sullivan M (1980). Incompatible crossmatch following negative antibody screen: Frequency and cause. Transfusion.

[6] Ambuja K, Shivaram C (2015). Red cell antibody screening in South India: lessons learnt. ISBT Sci Ser.

[7] (1998). Standards for Blood Banks and Transfusion Services.

[8] Pathak S, Chandrasekhar M, Wankhede GR (2011). Type and Screen policy in the blood bank: is AHG-XM still required? A study at a multispeciality corporate hospital in India. Asian J Transfus Sci.

[9] Chaudhary R, Agarwal N (2011). Safety of type and screen method compared to conventional antiglobulin crossmatch procedures for compatibility testing Indian setting. Asian J Transfus Sci.

[10] Masouredis SP (1982). Pretransfusion tests and compatibility: question of safety and efficacy. Blood.

[11] Keehan SP, Cuckler GA, Sisko AM, Madison AJ, Smith SD, Lizonitz JM (2012). National Health Expenditure Projections: modest annual growth until coverage expands and economic growth accelerates. *Health Aff* (Millwood).

[12] Varney SJ, Guest JF (2003). The annual cost of blood transfusions in the UK. Transfus Med.

[13] Pereira A (2005). Blood inventory management in the type and screen era. Int J Transfus Med.

[14] Office of Biologics Research and Review, National Centre for Drugs and Biologics, United States (1984). Equivalent methods for compatibility testing: memorandum to establishments.

[15] American Association of Blood Banks (AABB) (1978). Standards of blood banks and transfusion services.

[16] Schonwille H, Brand A (2005). Alloimmunization to red blood cell antigens after universal leucodepletion. A regional multicentre retrospective study. Br J Haematol.

[17] Singer ST, Wu V, Mignacca R, Kuypers FA, Morel P, Vichinsky EP (2000). Alloimmunization in transfusion dependent Thalassemia patients of predominantly Asian descent. Blood.

[18] Lee GK, Ma ESK, Tang M, Lam CCK (2003). Prevalence and specificity of clinically significant red cell alloantibodies in Chinese women during pregnancy: a review of cases from 1997 to 2001. Transfus Med.

[19] Zaman S, Chaurasia R, Chatterjie K, Thapliyal R (2014). Prevalence and specificity of RBC alloantibodies in Indian patients attending a tertiary care hospital. Adv Hematol.

[20] Makroo RN, Bhatia A, Hegde V, Chowdry M, Thakur UK, Rosamma NL (2014). Antibody screening and identification in the general population at a tertiary care hospital in New Delhi, India. Indian J Med Res.

[21] Agrawal A (2014). Type and Screen policy: is there any compromise on blood safety?. Transfus Apher Sci.

[22] Heddle NM, O’Hoski P, Singer J, Mc Bride JA, Ali MA, Kelton JG (1992). A prospective study to determine the safety of omitting the antihuman globulin crossmatch from pretransfusion testing. Br J Haematol.

[23] Armstrong B, Wilkinson R, Smart E (2008). Compatibility testing. ISBT Sci Ser.

[24] Davis SP, Baresso C, Ness PM (1983). Maximizing the benefits of Type and Screen by continued surveillance of transfusion practice. Am J Med Technology.

[25] Tiwari AK, Aggarwal G, Dara RC, Arora D, Gupta GK, Raina V (2017). First Indian study to establish safety of immediate spin crossmatch for red blood cell transfusion in antibody screen negative recipients. Asian J Transfus Sci.

